# The Protective Effect of *Polygonum cuspidatum* (PCE) Aqueous Extract in a Dry Eye Model

**DOI:** 10.3390/nu10101550

**Published:** 2018-10-19

**Authors:** Bongkyun Park, Ik Soo Lee, Soo-Wang Hyun, Kyuhyung Jo, Tae Gu Lee, Jin Sook Kim, Chan-Sik Kim

**Affiliations:** 1 Clinical Medicine Division, Korea Institute of Oriental Medicine, Daejeon 34054, Korea; bkpark@kiom.re.kr (B.P.); jopd7414@kiom.re.kr (K.J.); berong35@kiom.re.kr (T.G.L.); 2 Herbal Medicine Research Division, Korea Institute of Oriental Medicine, Daejeon 34054, Korea; knifer48@kiom.re.kr (I.S.L.); swhyun@kiom.re.kr (S.-W.H.); jskim@kiom.re.kr (J.S.K.); 3 Korean Medicine Life Science, University of Science Technology (UST), Daejeon 34113, Korea

**Keywords:** *Polygonum cuspidatum*, hyperosmolar stress, exorbital lacrimal gland-excised, dry eye, human corneal epithelial cells, inflammation, apoptosis, oxidative stress, matrix metallopeptidase 9 (MMP9), Mucin4

## Abstract

Dry eyes are caused by highly increased osmolarity of tear film, inflammation, and apoptosis of the ocular surface. In this study, we investigated the effect of *Polygonum cuspidatum* (PCE) aqueous extract in in vivo and in vitro dry eye models. Dry eye was induced by excision of the lacrimal gland and hyperosmotic media. In vivo, oral administration of PCE in exorbital lacrimal gland-excised rats recovered tear volume and Mucin4 (MUC4) expression by inhibiting corneal irregularity and expression of inflammatory cytokines. In vitro, hyperosmotic media induced human corneal epithelial cell (HCEC) cytotoxicity though increased inflammation, apoptosis, and oxidative stress. PCE treatment significantly inhibited expression of cyclooxygenase-2 and inflammatory cytokines (interleukin-6 and tumor necrosis factor-α), and activation of NF-κB p65 in hyperosmolar stress-induced HCECs. Hyperosmolarity-induced increase in Bcl-2-associated X protein (BAX) expression and activation of cleaved poly (ADP-ribose) polymerase and caspase 3 were attenuated in a concentration-dependent manner by PCE. PCE treatment restored anti-oxidative proteins such as heme oxygenase-1 (HO-1), superoxide dismutase-1 (SOD-1), and glutathione peroxidase (GPx) in hyperosmolar stress-induced HCECs. These data demonstrate that PCE prevents adverse changes in the ocular surface and tear fluid through inhibition of hyperosmolar stress-induced inflammation, apoptosis, and oxidation, suggesting that PCE may have the potential to preserve eye health.

## 1. Introduction

Dry eye disease (DED), known as keratoconjunctivitis sicca (KCS), is the most common disorder of the ocular surface and tears. Symptoms of DED are dryness, redness, light sensitivity, itching, and irritation [1]. Dry eyes are mainly caused by highly elevated osmolarity of tear film and inflammation of the ocular surface [2]. Tear film is well organized in the three kinds of layers, the glycocalyx layer, the intermediate aqueous layer, and the outmost tear film lipid layer. Each layer has the critical roles for eye health. The glycocalyx layer is viscous because it consists of many membrane-bound and secreted mucins. The intermediate aqueous layer includes a high concentration of soluble mucins. In addition, the outer lipid layer is made up of an outer non-polar lipid layer and inner polar lipid layer, distributing a smooth optical surface for the corneal and delaying moisture evaporation [3,4,5]. Therefore, decreased aqueous tear flow or abnormal evaporation of the aqueous phase causes hyperosmolarity of tears, a critical step towards severe DED pathology [6]. Previous studies have reported that hyperosmolarity is a potent stressor which stimulates inflammatory responses [7,8], induces apoptosis of corneal epithelial cells [9], and increases the expression of matrix metalloproteinases (MMPs) in the ocular epithelial surface [10,11]. Therefore, regulating the immune response and apoptosis are critical factors to maintain a stable and healthy ocular surface. Pharmacotherapies to treat DED are still limited. Most patients are treated with cyclosporine, the only approved pharmacotherapy thus far. However, cyclosporine, an immunosuppressant, has several adverse side effects: burning, pain or other discomfort of the eye, as well as bone marrow suppression, cystitis, and hypertension [12,13]. In addition, artificial tears only provide temporary relief and do not alter the chronic progressive course of DED [14,15].

*Polygonum cuspidatum* is a herbaceous perennial plant of the genus *Polygonum* found in Asia and North America [16]. It is used as a folk herbal medicine for cough, hepatitis, jaundice, amenorrhea, leucorrhoea, arthralgia, hyperlipidemia scalding and bruises, snake bites, and carbuncles [17]. The critical bioactive constituents of *P. cuspidatum* are polydatin, resveratrol, and anthraquinones such as emodin and its glycosides. It also includes flavonoids such as quercetin and (+)-catechin [18]. The leaves and roots of *P. cuspidatum* contain abundant amino acids, vitamins, and flavonoids that have anti-bacterial, anti-inflammatory, anti-oxidative, and wound-healing effects [19,20,21,22]. Ethanol extracts of *P. cuspidatum* inhibited hepatitis B virus (HBV) in a stable HBV-producing cell line through anti-inflammatory activity [23]. Many clinical studies have demonstrated that *P. cuspidatum* extract and its bioactive constituents have antimicrobial, anti-inflammatory, anti-virus, neuroprotective, and cardioprotective effects. However, the effects of *P. cuspidatum* aqueous extract (PCE) on hyperosmolarity-induced inflammation and apoptosis in human corneal epithelial cells and dry eye-induced rats have not been examined.

The purpose of this study was to estimate the protective effect of *Polygonum cuspidatum* (PCE) aqueous extract in a dry eye model as well as to demonstrate the underlying mechanisms.

## 2. Materials and Methods

### 2.1. Preparation of Polygonum Cuspidatum Extract

The aero component of *Polygonum cuspidatum* was provided by Samil. Co. Ltd. (Seoul, Korea). Briefly, *P. cuspidatum* (48 g) was extracted with the first distilled water at 100 °C for 3 h, and the extract was performed by spray-drying (yield: 10.5%). The PCE was standardized using the reference compounds, caftaric acid, polydatin, rutin, quercitrin, and resveratrol (Sigma, St. Louis, MO, USA), by high-performance liquid chromatography (HPLC) according to previously described protocols [24]. Briefly, the PCE (10 mg) was liquefied in 50% methanol (10 mL). The solution was use through a 0.2 μm filter paper (Millipore, Burlington, MA, USA) prior to injection. HPLC analysis was performed with an Agilent 1200 HPLC instrument (Agilent Technologies, Santa Clara, CA, USA). The column used was a Prontosil C18 (4.6 × 150 mm, 5.0 μm, Bischoff, Eltingen, Germany). The mobile phase consisted of 0.1% formic acid in water and acetonitrile. Column temperature was maintained at 40 °C. Analysis was performed at a flow rate of 1.0 mL/min for 50 min and monitored at 330 nm. The injection volume of the sample was 10 μL.

### 2.2. Animals and Treatment

Seven-week-old male Wistar rats were purchased from Orient Bio (Seoul, Korea). The dry eyed rat model was carried out according to previously described protocols [25]. Rats in the normal control group (NOR) were not carried out with any surgical operation. At 3 days after surgery, the operated rats were randomly allocated to four groups: (1) vehicle-treated dry eyed rats (DED); (2) 10 mg/kg PCE-treated DED rats (PCE-10); (3) 100 mg/kg PCE-treated DED rats (PCE-100); (4) 250 mg/kg PCE-treated DED rats (PCE-250). The animal experiments were allowed by the Institutional Animal Care and Use Committee (IACUC approval No. 18-028).

### 2.3. Tear Volume Measurement

Tear volume was performed at day 7 after surgical operation. All procedures of experiment carried out according to previously known protocols [25]. Phenol red-impregnated cotton threads (Zone Quick; FCI Ophthalmics, Pembroke, MA, USA) were used with forceps and located in the lateral canthus for 1 min. The tear volume was quantified under an optical microscope and showed in terms of the length of color-changed threads that absorbed the tear fluid.

### 2.4. Analysis of Corneal Irregularity

The changed Corneal smoothness was investigated in rats from each group as previously described [26]. In brief, reflected lines of ring-shaped light from the fiber-optic ring illuminator of a stereomicroscope (SZ51; Olympus, Tokyo, Japan) were lighted on the corneal surface after anesthesia, and the reflected lines of light were captured with a DP21 digital camera (Olympus). Scores of changed corneal smoothness were classified according to the number of distorted quadrants in the reflected white ring as follows: 0, no distortion; 1, distortion in one quadrant; 2, distortion in two quadrants; 3, distortion in three quadrants; 4, distortion in all four quadrants; 5, severe distortion in which no ring could be recognized

### 2.5. Immunohistochemistry (IHC)

To determine expression levels of Mucin4 (MUC4), IHC was conducted as previously indicated [27] In brief, the rat anti-MUC4 antibody was used (Cell Signaling, Danvers, MA, USA). To measure MUC4, the slides were marked with a LSAB kit (DAKO, Santa Clara, CA, USA) and specified with a DAB substrate kit (DAKO). To analysis shapes, the immunoreactive intensity per unit area (mm^2^) was examined using ImageJ software (NIH, Bethesda, MD, USA).

### 2.6. Human Corneal Epithelial Cell (HCEC) Culture

The HCECs were purchased from the American Type Culture Collection (ATCC) (Manassas, VA, USA). Cells were maintained in ATCC Mammary Epithelial Cell Basal Medium (Manassas, VA, USA) containing ATCC Mammary Epithelial Cell Growth Kit (Manassas, VA, USA). HCECs were incubated at 37 °C/5% CO_2_, and the medium was exchanged daily. The subculture was carried out when the cell layers were confluent (2–3 days).

### 2.7. Cell Viability Assay

The effect of PCE on cell viability was determined by Cell Counting Kit-8 (CCK-8) (Dojindo Molecular Technologies, Rockville, MD, USA). Human corneal epithelial cells (HCECs) (2 × 10^4^ cells/well) were seeded in a 96-well cell culture plate and treated with PCE for 24 h. After 24 h, 20 μL CCK-8 solution was added and incubated for a further 4 h. After 4 h, the absorbance was measured at 450 nm using an microplate reader device, Infinite-M200 spectrophotometer (Tecan, Männedorf, Switzerland).

### 2.8. Western Blot Analysis

HCECs were seeded in 60 mm dishes (6 × 10^5^ cells/mL) and co-treated with PCE (1, 10, 100 μg/mL) for 1 or 24 h. The cells were washed with phosphate-buffered saline (PBS) and lysed with RIPA lysis buffer (Invitrogen, Carlsbad, CA, USA). Cell lysate (30 mg) was separated by 8, 10, and 12% sodium dodecyl sulfate polyacrylamide gel electrophoresis (SDS-PAGE) gels and transferred to Polyvinylidene fluoride (PVDF) or nitrocellulose membranes. The membranes were blocked in skim milk dissolved in Tris-buffered saline with 0.1% Tween 20 (TBST) buffer for 1 h. Membranes were incubated with the following diluted (1:1000) primary antibodies: Cyclooxygenase-2 (COX-2), p65, phopho-p65, Poly (ADP-ribose) polymerase (PARP), Caspase-3, Bcl-2, Bcl-2-associated X protein (BAX), HO-1, Superoxide dismutase-1 (SOD-1), glutathione peroxidase (GPx) β-actin, and Lamin A (Cell Signaling, Danvers, MA, USA) in Tris-HCl-based buffer containing 0.2% Tween 20 (TBS-T; pH 7.5). Band intensities were measured using ImageJ software (NIH, Bethesda, MD, USA).

### 2.9. Nuclear and Cytosol Protein Extraction

Proteins derived the nucleus and cytosol were isolated using NE-PER Nuclear and Cytoplasmic Extraction Reagents (#78835, Thermo Scientific, Waltham, MA, USA). In brief, HCECs were collected with trypsin-EDTA and washed with PBS. Then, the collected cells were centrifuged at 15,000 rpm for 10 min, and the supernatants were eliminated. Ice-cold CER-I and -II solutions were added following the manufacturer’s instructions to isolate the cytoplasmic proteins from the nuclear compartment proteins. Western blotting for p65, Lamin A, and β-actin was conducted.

### 2.10. Quantitative Realtime-Polymerase Chain Reaction (PCR)

Total RNA was extracted using a single-step guanidinium thiocyanate-phenol-chloroform method. The yield and purity of the RNA were confirmed by measuring the ratio of the absorbance at 260 and 280 nm. Isolated RNA (1 mg/mL) was reverse transcribed using a SuperScript II kit for cDNA. The cDNA was subjected to quantitative realtime (qRT)-PCR using specific primers listed in Table 1 by thermocyclers. (Bio-Rad, Hercules, CA, USA).

### 2.11. Statistical Analysis

All representative data from three independent experiments are demonstrated as means ± standard error of the mean (SEM). The one-way ANOVA was performed to compare between control and experimental values by Prism 7 from Graphpad Software (San Diego, CA, USA). Statistical significance was defined as *p* < 0.05.

## 3. Results

### 3.1. Polygonum Cuspidatum Active Compound Isolation and Determination

**Figure 1:**
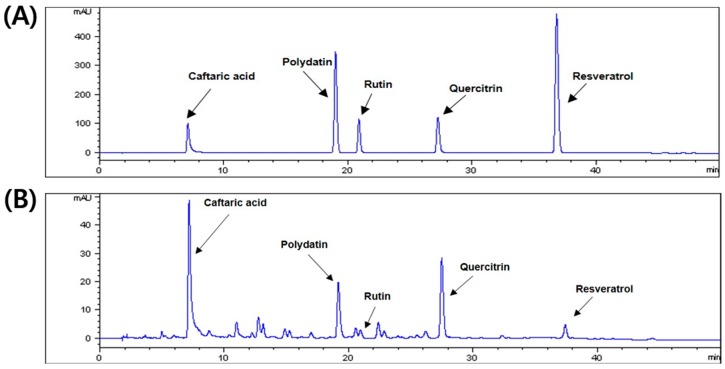
High-performance liquid chromatography (HPLC) chromatogram of *Polygonum cuspidatum* aqueous extract. (**A**) The five standard components and (**B**) aqueous extract of *P. cuspidatum* at 330 nm.

To confirm the quality of PCE, the HPLC-detector was used to analyze the active compounds in PCE. The UV detector was set at 330 nm for HPLC analysis of five standard compounds: caftaric acid, polydatin, rutin, quercitrin, and resveratrol. As shown in Figure 1A,B, we investigated the retention times and UV spectral data of PCE compared to those of standard compounds by HPLC analysis. Peaks 1–5 of PCE were recognized as caftaric acid, polydatin, rutin, quercitrin, and resveratrol, respectively. Compared to these compounds and PCE, PCE also contained five compounds and demonstrated the same retention times.

### 3.2. Effects of PCE on Tear Production

**Figure 2:**
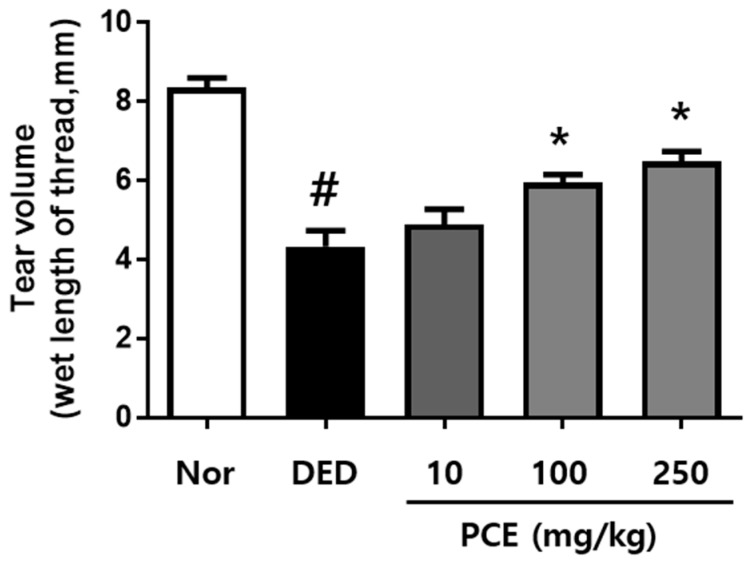
Effects of *P. cuspidatum* (PCE) on tear production in exorbital lacrimal gland-excised rats. Tear volume was determined using the phenol red thread tear test. Tear volume was revealed in mm of thread that became wet by tears and turned red. Normal control (NOR): normal control mice, Dry eye disease (DED): vehicle-treated dry eye rats, PCE-10: 10 mg/kg PCE-treated DED rats, PCE-100: 100 mg/kg PCE-treated DED rats, and PCE-250: 250 mg/kg PCE-treated DED rats. The values in the bar graph represent the mean ± standard error (SE), *n* = 10. # *p* < 0.05, significantly different from normal rats. * *p* < 0.05, significantly different from vehicle-treated dry eye rats.

To examine the effects of PCE on tear production, we used an exorbital lacrimal gland-excised model of DED. As shown in Figure 2, the excised lacrimal gland group (DED) (4.33 ± 1.28 mm, *p* < 0.0001) demonstrated significantly inhibited tear fluid secretion compared to that of the normal group (8.36 ± 0.76 mm). The tear volumes of the groups orally administrated with PCE (100 and 250 mg/kg) significantly increased to 5.95 ± 0.71 and 6.49 ± 0.89 mm (*p* < 0.05), respectively, after treatment for 5 days compared to those of the DED group.

### 3.3. Effects of PCE on Corneal Surface Irregularities

**Figure 3:**
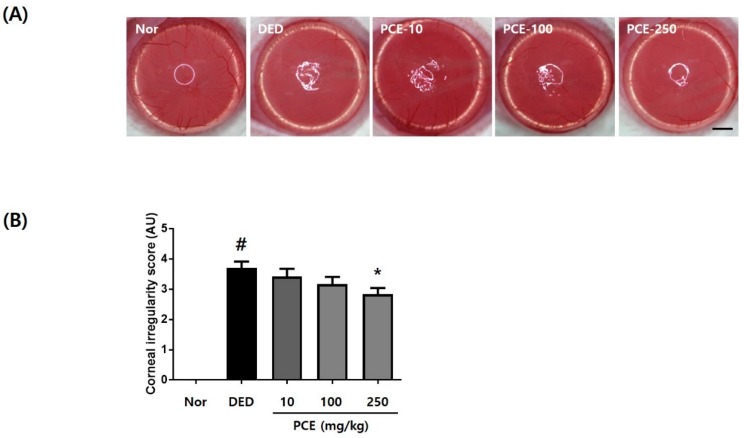
Effects of *P. cuspidatum* (PCE) on corneal surface irregularities in exorbital lacrimal gland-excised rats. (**A**) Reflected images of a white ring from the fiber-optic ring illuminator of a stereomicroscope. Scale bar is 1 mm. (**B**) Corneal irregularity was graded according to the number of distorted quarters in the reflected white ring as follows: 0, no distortion; 1, distortion in one quarter; 2, distortion in two quarters; 3, distortion in three quarters; 4, distortion in all four quarters; 5, severe distortion, in which no ring could be recognized. The values in the bar graph represent the mean ± SEM, *n* = 10. # *p* < 0.05, significantly different from normal rats, * *p* < 0.05, significantly different from vehicle-treated dry eye rats.

Excision of the lacrimal gland gradually increased the corneal surface irregularity in all groups (Figure 3A). Compared to the DED group, the group that received oral administration of PCE (250 mg/kg) demonstrated a circular white ring 5 days later. The quantitative results of corneal irregularity score are indicated in Figure 3B. Corneal irregularity scores following exorbital lacrimal gland excision significantly increased to 3.7 ± 0.67 (*p* < 0.05) compared to those of the normal group. Oral administration of PCE (250 mg/kg) significantly attenuated the corneal irregularity score to 2.83 ± 0.2 (*p* < 0.05) compared to that of the DED group, suggesting that PCE may help to maintain tear film integrity.

### 3.4. Effects of PCE on MUC4 Expression and Inflammatory Cytokines

**Figure 4:**
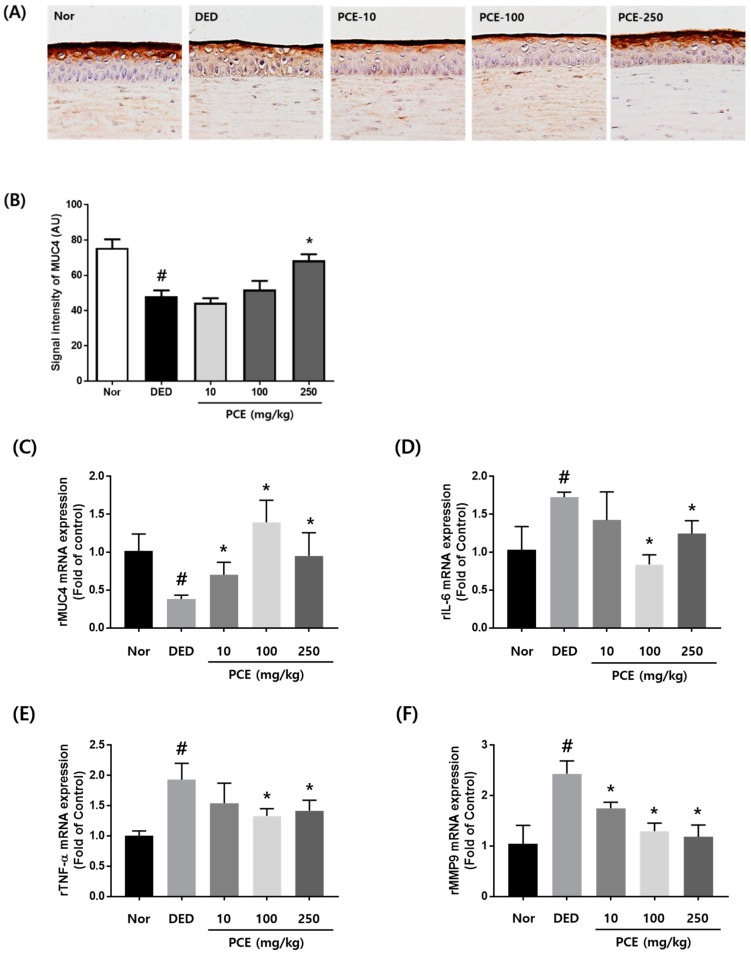
Effects of *P. cuspidatum* (PCE) on expression of mucin 4 (MUC4) and inflammatory cytokines in exorbital lacrimal gland-excised rats. (**A**) Immunohistochemical staining for Muc4. (**B**) Morphometric analysis of Muc4-positive signal density in corneal sections from each group. The values in the bar graph represent the mean ± SEM, *n* = 10. RNA was extracted from the corneal tissue of exorbital lacrimal gland-excised rats. mRNA levels of (**C**) *MUC4*, (D) *IL-6*, (**E**) *TNF-α*, and (**F**) *MMP9* were assessed by real-time PCR assay. *GAPDH* was considered an internal control. Data are the mean ± SEM of three independent experiments for all groups. # *p* < 0.05, significantly different from normal mice, * *p* < 0.05, significantly different from vehicle-treated dry eye mice.

Mucins have important roles in maintaining the environment of the eyes. In particular, mucins keep the ocular surface wet and prevent adverse environmental conditions [28]. Therefore, loss of mucins creates a dry eye environment. We investigated whether PCE affected the restoration of mucin production in exorbital lacrimal gland-excised rats. As shown in Figure 4A, the expression of MUC4 was significantly decreased in the DED group compared to that in the normal group. However, the group orally administered with PCE (250 mg/kg) exhibited restored MUC4 expression and signal intensity compared to that of the DED group (Figure 4A,B). Next, we evaluated the effects of PCE on mRNA expression of inflammatory genes, *MMP9* and *MUC4*, in corneal tissue derived from dry eyed rats. *MUC4* mRNA expression was significantly inhibited in the DED group. In contrast, inhibited *MUC4* mRNA expression was recovered in a dose-dependent manner of PCE (Figure 4C). In the DED group, *IL-6*, *TNF-α*, and *MMP9* mRNA expression was increased by 74%, 96%, and 143%, respectively. In the groups orally administered with PCE (100 and 250 mg/kg), *IL-6* and *TNF-α* mRNA expression was significantly attenuated, and *MMP9* mRNA expression was inhibited in a dose-dependent manner with PCE treatment, suggesting that PCE restored MUC4 expression in corneal tissue by inhibiting inflammatory cytokines and MMP9 (Figure 4D–F).

### 3.5. Effects of PCE on Hyperosmolar Stress-Induced Cell Death

**Figure 5:**
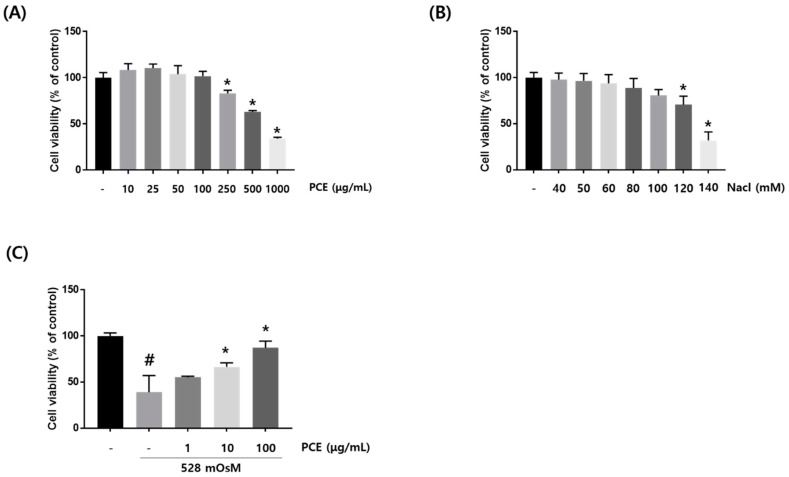
Effects of *P. cuspidatum* (PCE) on hyperosmolar stress-induced cell death in human corneal epithelial cells (HCECs). (**A**,**B**) HCECs were treated with PCE or NaCl at different concentrations for 24 h. (**C**) HCECs were co-treated with PCE and hyperosmotic media for 24 h. To investigate cell viability, CCK-8 (Cell Counting Kit-8) assay was performed. Data are the mean ± SEM of three independent experiments for all groups. # *p* < 0.05, significantly different from untreated group, * *p* < 0.05, significantly different from hyperosmotic-treated group.

To investigate the effect of PCE on cytotoxicity in HCECs, we performed CCK-8 assay at the indicated PCE concentration for 24 h. Cell viability showed that PCE attenuated cell toxicity at concentrations from 250 μg/mL (Figure 5A). To examine the effect of hyperosmolar stress-induced cytotoxicity in HCECs, we used hyperosmolar cell culture media by adding sodium chloride (NaCl) into HCEC culture media at the indicated NaCl concentration for 24 h. Hyperosmolar cell culture media significantly increased cytotoxicity at 120 mM NaCl (Figure 5B). Osmolarity analysis revealed that cell culture media containing 120 mM NaCl had an osmolarity of 528 mOsM. This informed our choice of PCE concentrations (1, 10, and 100 μg/mL) and hyperosmotic cell culture media (528 mOsM) for subsequent experiments. To investigate the protective effects of PCE on cell viability in hyperosmotic-stimulated HCECs, we used CCK-8 assay in the co-treatment with the indicated PCE concentrations (1, 10, and 100 μg/mL) and hyperosmotic cell culture media for 24 h. CCK-8 assay revealed that cell viability was significantly recovered by PCE at concentrations of 10 and 100 μg/mL (Figure 5C).

### 3.6. Effects of PCE on Inflammation

**Figure 6:**
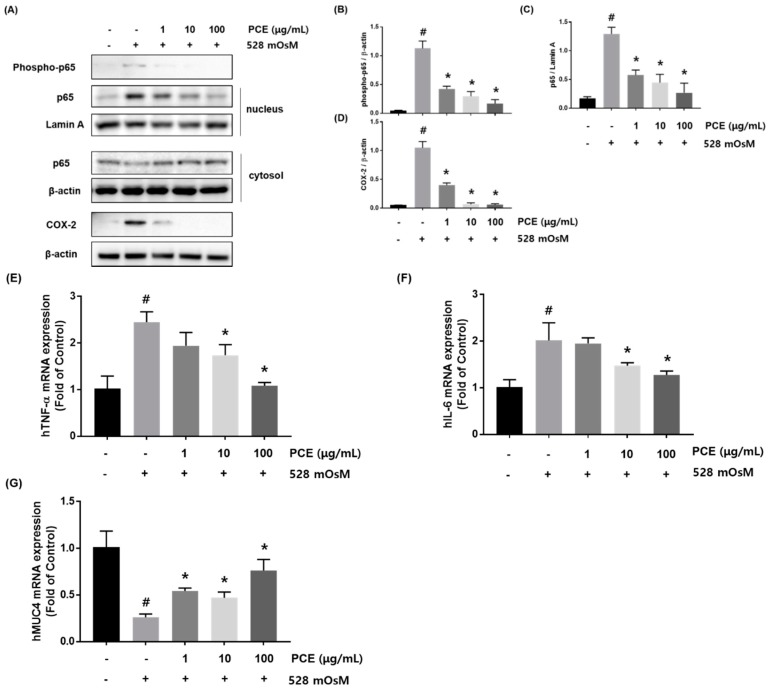
Effects of *P. cuspidatum* (PCE) on inflammation in hyperosmolar stress-stimulated human corneal epithelial cells (HCECs). (**A**) HCECs were co-treated with the indicated concentrations of PCE and hyperosmotic media (528 mOsM) for 1 h or 8 h. The protein expression of phosphor-p65 and COX-2 (Cyclooxygenase-2) was analyzed by western blot analysis. Cytoplasmic and nuclear levels of NF-κB p65 were detected by western blotting to analyze the translocation of NF-κB. β-actin and Lamin A were used as loading controls. (**B**–**D**) The relative intensities are expressed as the ratio of phosphor-p65, nuclear p65, and COX-2 to β-actin or Lamin A. RNA was extracted from hyperosmolar stress-stimulated HCECs, and the mRNA levels of (**E**) *TNF-α*, (**F**) *IL-6*, and (**G**) *MUC4* were assessed by real-time PCR assay. *GAPDH* was considered an internal control. Data are the mean ± SEM of three independent experiments for all groups. # *p* < 0.05, significantly different from untreated group, * *p* < 0.05, significantly different from hyperosmotic-treated group.

Hyperosmotic stress induces the production of inflammatory cytokines in the ocular surface [29]. To determine whether PCE inhibited inflammation in hyperosmotic-stimulated HCECs, the protein expression of phosphor-p65 and COX-2 was examined. Phosphor-p65 and COX-2 expression were significantly increased by hyperosmotic stress. However, treatment with PCE markedly inhibited the expression of phosphor-p65 and COX-2 in a concentration dependent manner (Figure 6A). In addition, we investigated the effect of PCE on hyperosmotic stress-induced activation of NF-κB. HCECs were co-treated with the indicated concentrations of PCE (1, 10, and 100 μg/mL) and hyperosmotic cell culture media for 1 h. Western blot analysis demonstrated that the expression of nuclear p65, known to control transcription of inflammatory genes, was significantly decreased by PCE in a concentration-dependent manner, suggesting that treatment with PCE resulted in attenuation of hyperosmotic stress-induced nuclear translocation of NF-κB (Figure 6A). We next examined whether PCE affected the expression of inflammatory cytokines and MUC4. HCECs were co-incubated with the indicated concentrations of PCE and hyperosmotic media for 24 h. mRNA expression of *TNF-α* and *IL-6* was increased by treatment with hyperosmotic media, but PCE suppressed hyperosmolar stress-induced *TNF-α* and *IL-6* mRNA expression in a concentration-dependent manner (Figure 6E,F). The decrease in *MUC4* mRNA expression by hyperosmolar stress was significantly restored by PCE treatment (Figure 6G).

### 3.7. Effects of PCE on Hyperosmolar Stress-Induced Apoptotic Cell Death

**Figure 7:**
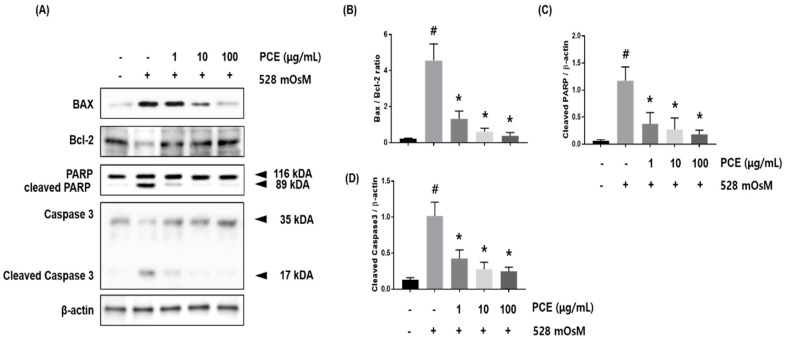
Effects of *P. cuspidatum* (PCE) on hyperosmolar stress-induced apoptotic cell death in human corneal epithelial cells (HCECs). HCECs were co-treated with the indicated concentrations of PCE and hyperosmotic media for 24 h. (**A**) The protein expression of BAX, Bcl-2, Caspase 3, and polymerase (PARP) was analyzed by western blot analysis. β-actin was used as a loading control. (**B**–**D**) The relative intensities are expressed as the ratio of BAX, Bcl-2, cleaved PARP, and cleaved caspase 3 to β-actin. Data are the mean ± SEM of three independent experiments for all groups. # *p* < 0.05, significantly different from untreated group, * *p* < 0.05, significantly different from hyperosmotic-treated group.

A variety of apoptosis pathways are associated with hyperosmolar stress-induced ocular surface damage including the extrinsic pathway triggered by death receptors and intrinsic mitochondria-related pathway [9,30]. The intrinsic apoptotic pathway may be initiated by hyperosmolar stress, causing elevated BAX expression which attenuates Bcl-2 expression. Activation of Poly (ADP-ribose) polymerase (PARP) and caspase 3 is induced by hyperosmolar stress, suggesting that inhibition of these mediators may decrease hyperosmolar stress-induced cell death [31]. We investigated whether PCE had the ability to protect against hyperosmolar stress-induced apoptosis in HCECs. Hyperosmolar stress induced apoptotic cell death in HCECs through upregulation of BAX and activation of PARP and caspase 3. PCE treatment significantly inhibited BAX expression, activation of PARP, and Caspase3; and recovered expression of Bcl-2 in a concentration-dependent manner. These findings revealed that PCE had protective effects against hyperosmolar stress-induced intrinsic apoptotic pathway activation in HCECs (Figure 7).

### 3.8. Effects of PCE on Hyperosmolar Stress-Induced Oxidative Stress

**Figure 8:**
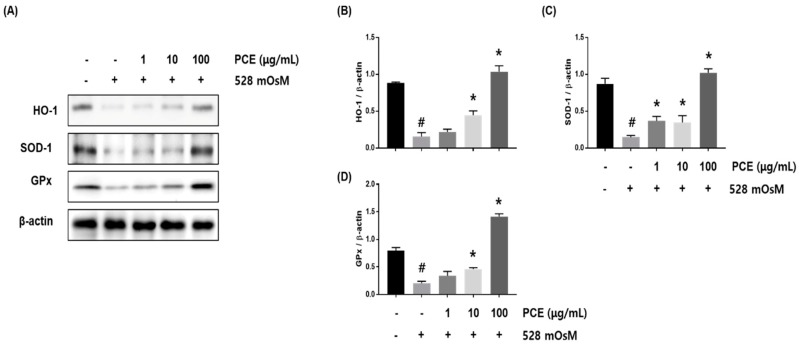
Effects of *P. cuspidatum* (PCE) on oxidative stress in hyperosmolar stress-induced human corneal epithelial cells (HCECs). HCECs were co-treated with the indicated concentrations of PCE and hyperosmotic media for 24 h. (**A**) The protein expression of HO-1, SOD-1, and GPx was analyzed by western blot analysis. β-actin was used as a loading control. (**B**–**D**) The relative intensities are expressed as the ratio of HO-1, SOD-1, and GPx to β-actin. Data are the mean ± SEM of three independent experiments for all groups. Means unlike letters in a column with differ significantly (*p* < 0.05) # *p* < 0.05, significantly different from untreated group, * *p* < 0.05, significantly different from hyperosmotic-treated group.

To assess whether PCE decreased oxidative stress in hyperosmolar stress-induced HCECs, HCECs were co-treated with PCE and hyperosmotic media at the indicated concentrations for 24 h. Western blot analysis revealed that hyperosmolar stress significantly inhibited the expression of HO-1, SOD-1, and GPx in HCECs. The decreased expression of HO-1, SOD-1, and GPx was restored by PCE treatment in a concentration-dependent manner, indicating that PCE may inhibit oxidative stress (Figure 8).

## 4. Discussion

In the present study, we investigated the effects of PCE on DED in exorbital lacrimal gland-excised rats and hyperosmolar stress-induced HCECs. These data demonstrated that the recovery of tear volume, corneal irregularity, MUC4 expression, and anti-oxidative genes by PCE may be helpful in therapy for DED. In vivo, PCE recovered tear volume and MUC4 expression by inhibiting corneal irregularity and mRNA expression of inflammatory cytokines. In vitro, PCE inhibited inflammation, apoptosis, and oxidative stress in hyperosmolar stress-induced HCECs via attenuation of COX-2 expression, nuclear translocation of NF-κB, production of inflammatory cytokines, Bax expression, and activation of PARP and caspase 3.

The ocular surface comprises the conjunctiva, cornea, lacrimal glands, related tear and connective tissue, eyelashes, and eyelids; these involve the ocular epithelia, nervous and endocrine systems, immune responses and vasculature [32] Additionally, homeostasis of the ocular surface system is affected by tear film stability and osmolarity leading to hyperosmotic damage such as oxidation, inflammation, and apoptosis [33] Several animal models of DED have been developed to evaluate the pathology of dry eyes and the efficacy of therapeutic candidates. Recently, lacrimal gland excision has been used as an experimental model to induce DED though attenuated tear volume, increased corneal surface irregularity, disrupted corneal epithelial barrier function, and reduced conjunctival goblet cell viability [25,34,35,36]. In our study, lacrimal gland excision significantly increased the production of pro-inflammatory cytokines such as IL-6 and TNF-α, and MMP9 expression, but decreased MUC4 expression in corneal epithelial tissue. Malfunction of lacrimal glands changes the balance of tear film constituents and promotes inflammation on the ocular surface by several mechanisms [37]. First, there is reduced production of anti-inflammatory factors such as lactoferrin [38]. Second, pro-inflammatory cytokines (IL-6 and TNF-α) and proteolytic enzymes (MMPs) are increased by stressed ocular surface, glandular epithelial cells, and inflammatory cells that penetrate these tissues. Third, latent inactive cytokines and proteases are activated by ocular surface infection and wounds [39,40]. In the present study, oral administration of PCE inhibited mRNA expression of inflammatory cytokines in exorbital lacrimal gland-excised rats. In addition, treatment with PCE in hyperosmolar stress-induced HCECs significantly attenuated COX-2 expression and mRNA expression of *IL-6* and *TNF-α* through suppression of NF-κB activation, suggesting that PCE has potent anti-inflammatory activity.

Another pathologic alteration in DED is increased activity and concentration of MMPs [41,42]. In the case of MMP9, this enzyme lyses the various components of the corneal epithelial basement membrane and tight junction proteins such as ZO-1 and occludin to maintain corneal epithelial barrier function [43,44]. Increased MMP9 activity in DED is related to a dysfunctional epithelial barrier, increased corneal epithelial erosions, and corneal surface irregularity [45,46] Our findings revealed that PCE inhibited the increase in *MMP9* mRNA expression in DED.

At the ocular surface, three types of mucins exist: the large gel-forming mucin (MUC5AC) in conjunctival goblet cells, the small soluble mucin MUC7 in the lacrimal gland acini, and the membrane-associated mucins MUCs 1, 4, and 16 in the corneal and conjunctival epithelia [47]. All mucins are hydrophilic and play roles in preservation of moisture on the eye surface. Therefore, the regulation and alteration of mucins in the ocular surface can lead to disease. In this study, MUC4 was significantly inhibited by excision of the lacrimal gland and hyperosmolarity. However, PCE significantly restored *MUC4* mRNA expression in vivo and in vitro, indicating that PCE may help to maintain the ocular surface of the corneal epithelium.

The major active compounds in PCE are caftaric acid, polydatin, rutin, quercitrin, and resveratrol. The five active compounds are present in many natural plant extracts, and previous studies have demonstrated that they have anti-inflammatory and anti-oxidative properties [48,49,50]. Therefore, PCE may promote anti-oxidative activity in DED by recovering expression of anti-oxidant proteins such as HO-1, SOD-1, and GPx in hyperosmolar stressed HCECs. We determined that polydatin was a major active component. Polydatin is a stilbenoid glucoside and a major resveratrol derivative [51]. Previous studies have shown that polydatin has many pharmacological functions including anti-oxidant and anti-inflammatory activity, and is used to treat chronic bronchitis, hepatitis, shock [52], and inflammatory processes of chronic pelvic pain in patients [53]. However, the effects of polydatin on DED in vivo and in vitro are unclear. Thus, further studies are required to identify the effects of polydatin on DED and the underlying mechanisms.

## 5. Conclusions

In conclusion, the present study showed that excision of lacrimal glands and hyperosmolarity induced changes such as decreased tear fluid, MUC4 expression, and severe corneal irregularity in the ocular surface by increasing inflammation. Theses alterations were rescued by PCE treatment. In vitro, PCE protected against hyperosmolar stress-induced inflammation, apoptosis, and oxidation by inhibiting expression of COX-2, BAX, MMP9; activation of NF-κB, caspase 3, and PARP; and increasing expression of MUC4 and anti-oxidative proteins. Overall, our data provide insight into the protective effects of PCE as a candidate for eye health.

## Figures and Tables

**Table 1:** Realtime PCR (polymerase chain reaction) primer sequences.

Genes	Sequence	
*rMuc4*	F	5′-CGGATTCCTTCTACGTTACAG-3′
	R	5′-GAATCGATCTGGACGGTACTTC-3′
*rMuc4*	F	5′-AGAGACTTCCAGCCAGTTGC-3′
	R	5′-AGCCTCCGACTTGTGAAGTG-3′
*rMuc4*	F	5′-TCGTCTACTCCTCAGAGCCC-3′
	R	5′-ACTTCAGCGTCTCGTGTGTT-3′
*rMMP9*	F	5′-CGCTTGGATAACGAGTTCTCTC-3′
	R	5′-GCAGGAGGTCATAGGTCACG-3′
*rGAPDH*	F	5′-CGTGAAAAGATGACCCAGAT-3′
	R	5′-ACCCTCATAGATGGGCACA-3′
*hMuc4*	F	5′-GCCCAAGCTACAGTGTGACTCA-3′
	R	5′-ATGGTGCCGTTGTAATTTGTTGT-3′
*hIL-6*	F	5′-AAATTCGGTACATCCTCGAC-3′
	R	5′-CAGGAACTGGATCAGGACTT-3′
*hTNF-α*	F	5′-TTCTCCTTCCTGCTTGTG-3′
	R	5′-CTGAGTGTGAGTGTCTGG-3′
*hMMP9*	F	5′-GGGACGCAGACATCGTCATC-3′
	R	5′-TCGTCATCGTCGAAATGGGC-3′
*hGAPDH*	F	5′-CCAGCCGAGCCACATCGCTC-3′'
	R	5′-ATGAGCCCCAGCCTTCTCCAT-3′

## References

[1] Stevenson W., Chauhan S.K., Dana R. (2012). Dry eye disease: An immune-mediated ocular surface disorder. Arch. Ophthalmol..

[2] Deng R., Su Z., Hua X., Zhang Z., Li D.-Q., Pflugfelder S.C. (2014). Osmoprotectants suppress the production and activity of matrix metalloproteinases induced by hyperosmolarity in primary human corneal epithelial cells. Mol. Vis..

[3] Lemp M.A. (2008). Advances in understanding and managing dry eye disease. Am. J. Ophthalmol..

[4] Nakamura T., Hata Y., Nagata M., Yokoi N., Yamaguchi S., Kaku T., Kinoshita S. (2015). JBP485 promotes tear and mucin secretion in ocular surface epithelia. Sci. Rep..

[5] Green-Church K.B., Butovich I., Willcox M., Borchman D., Paulsen F., Barabino S., Glasgow B.J. (2011). The international workshop on meibomian gland dysfunction: Report of the subcommittee on tear film lipids and lipid–protein interactions in health and disease. Investig. Ophthalmol. Vis. Sci..

[6] Bhavsar A.S., Bhavsar S.G., Jain S.M. (2011). A review on recent advances in dry eye: Pathogenesis and management. Oman J. Ophthalmol..

[7] Li D.-Q., Luo L., Chen Z., Kim H.-S., Song X.J., Pflugfelder S.C. (2006). JNK and ERK MAP kinases mediate induction of IL-1β, TNF-α and IL-8 following hyperosmolar stress in human limbal epithelial cells. Exp. Eye Res..

[8] De Paiva C.S., Corrales R.M., Villarreal A.L., Farley W.J., Li D.-Q., Stern M.E., Pflugfelder S.C. (2006). Corticosteroid and doxycycline suppress MMP-9 and inflammatory cytokine expression, MAPK activation in the corneal epithelium in experimental dry eye. Exp. Eye Res..

[9] Luo L., Li D.-Q., Pflugfelder S.C. (2007). Hyperosmolarity-induced apoptosis in human corneal epithelial cells is mediated by cytochrome c and MAPK pathways. Cornea.

[10] Li D., Pflugfelder S. (2005). Matrix Metalloproteinases in Corneal Inflammation. Ocul. Surf..

[11] Li D.-Q., Chen Z., Song X.J., Luo L., Pflugfelder S.C. (2004). Stimulation of matrix metalloproteinases by hyperosmolarity via a JNK pathway in human corneal epithelial cells. Investig. Ophthalmol. Vis. Sci..

[12] Melnikov S., Mayan H., Uchida S., Holtzman E.J., Farfel Z. (2011). Cyclosporine metabolic side effects: Association with the WNK4 system. Eur. J. Clin. Investig..

[13] Hornbeak D.M., Thorne J.E. (2015). Immunosuppressive therapy for eye diseases: Effectiveness, safety, side effects and their prevention. Taiwan J. Ophthalmol..

[14] Perry H.D., Solomon R., Donnenfeld E.D., Perry A.R., Wittpenn J.R., Greenman H.E., Savage H.E. (2008). Evaluation of topical cyclosporine for the treatment of dry eye disease. Arch. Ophthalmol..

[15] Dastjerdi M.H., Hamrah P., Dana R. (2009). High-frequency topical cyclosporine 0.05% in the treatment of severe dry eye refractory to twice-daily regimen. Cornea.

[16] Zhang H., Li C., Kwok S.-T., Zhang Q.-W., Chan S.-W. (2013). A review of the pharmacological effects of the dried root of Polygonum cuspidatum (Hu Zhang) and its constituents. Evid.-Based Complement. Altern. Med..

[17] Zhu Y.-P., Woerdenbag H.J. (1995). Traditional Chinese herbal medicine. Pharm. World Sci..

[18] Zhang H., Zhang Q.-W., Wang L., Zhang X.-Q., Ye W.-C., Wang Y.-T. (2012). Two new anthraquinone malonylglucosides from Polygonum cuspidatum. Nat. Prod. Res..

[19] Kim Y.-S., Hwang C.-S., Shin D.-H. (2005). Volatile constituents from the leaves of Polygonum cuspidatum S. et Z. and their anti-bacterial activities. Food Microbiol..

[20] Bralley E.E., Greenspan P., Hargrove J.L., Wicker L., Hartle D.K. (2008). Topical anti-inflammatory activity of Polygonum cuspidatum extract in the TPA model of mouse ear inflammation. J. Inflamm..

[21] Hsu C.-Y., Chan Y.-P., Chang J. (2007). Antioxidant activity of extract from Polygonum cuspidatum. Biol. Res..

[22] Wu X.-B., Luo X.-Q., Gu S.-Y., Xu J.-H. (2012). The effects of Polygonum cuspidatum extract on wound healing in rats. J. Ethnopharmacol..

[23] Chang J.-S., Liu H.-W., Wang K.-C., Chen M.-C., Chiang L.-C., Hua Y.-C., Lin C.-C. (2005). Ethanol extract of Polygonum cuspidatum inhibits hepatitis B virus in a stable HBV-producing cell line. Antivir. Res..

[24] Lv W.-F., Ding M.-Y., Zheng R. (2005). Isolation and quantitation of amygdalin in Apricot-kernel and Prunus Tomentosa Thunb. by HPLC with solid-phase extraction. J. Chromatogr. Sci..

[25] Kim C.-S., Jo K., Lee I.-S., Kim J. (2016). Topical application of apricot kernel extract improves dry eye symptoms in a unilateral exorbital lacrimal gland excision mouse. Nutrients.

[26] Yoon K.-C., Ahn K.-Y., Choi W., Li Z., Choi J.-S., Lee S.-H., Park S.-H. (2011). Tear production and ocular surface changes in experimental dry eye after elimination of desiccating stress. Investig. Ophthalmol. Vis. Sci..

[27] Sohn E.J., Kim C.-S., Kim Y.S., Jung D.H., Jang D.S., Lee Y.M., Kim J.S. (2007). Effects of magnolol (5, 5′-diallyl-2, 2′-dihydroxybiphenyl) on diabetic nephropathy in type 2 diabetic Goto–Kakizaki rats. Life Sci..

[28] Corrales R.M., Narayanan S., Fernández I., Mayo A., Galarreta D.J., Fuentes-Páez G., Chaves F.J., Herreras J.M., Calonge M. (2011). Ocular mucin gene expression levels as biomarkers for the diagnosis of dry eye syndrome. Investig. Ophthalmol. Vis. Sci..

[29] Luo L., Li D.-Q., Corrales R.M., Pflugfelder S.C. (2005). Hyperosmolar saline is a proinflammatory stress on the mouse ocular surface. Eye Contact Lens.

[30] Khandekar N., Willcox M.D., Shih S., Simmons P., Vehige J., Garrett Q. (2013). Decrease in hyperosmotic stress–induced corneal epithelial cell apoptosis by l-carnitine. Mol. Vis..

[31] Bell L.M., Leong M.L., Kim B., Wang E., Park J., Hemmings B.A., Firestone G.L. (2000). Hyperosmotic stress stimulates promoter activity and regulates cellular utilization of the serum and glucocorticoid inducible protein kinase (Sgk) by a p38/MAPK dependent pathway. J. Biol. Chem..

[32] Gipson I.K. (2007). The ocular surface: The challenge to enable and protect vision: The Friedenwald lecture. Investig. Ophthalmol. Vis. Sci..

[33] Gipson I.K., Argueso P., Beuerman R., Bonini S., Butovich I., Dana R., Dartt D., Gamache D., Ham B., Jumblatt M. (2007). Research in dry eye: Report of the Research Subcommittee of the International Dry Eye WorkShop (2007). Ocul. Surf..

[34] Kurose M., Meng I.D. (2013). Dry eye modifies the thermal and menthol responses in rat corneal primary afferent cool cells. J. Neurophysiol..

[35] Meng I.D., Barton S.T., Mecum N.E., Kurose M. (2015). Corneal sensitivity following lacrimal gland excision in the rat. Investig. Ophthalmol. Vis. Sci..

[36] Stevenson W., Chen Y., Lee S.-M., Lee H.S., Hua J., Dohlman T., Shiang T., Dana R. (2014). Extraorbital lacrimal gland excision: A reproducible model of severe aqueous tear-deficient dry eye disease. Cornea.

[37] Pflugfelder S.C. (2004). Antiinflammatory Therapy for Dry Eye.

[38] McCollum C.J., Foulks G.N., Bodner B., Shepard J., Daniels K., Gross V., Kelly L., Cavanagh H.D. (1994). Rapid assay of lactoferrin in keratoconjunctivitis sicca. Cornea.

[39] Solomon A., Dursun D., Liu Z., Xie Y., Macri A., Pflugfelder S.C. (2001). Pro-and anti-inflammatory forms of interleukin-1 in the tear fluid and conjunctiva of patients with dry-eye disease. Investig. Ophthalmol. Vis. Sci..

[40] Afonso A.A., Sobrin L., Monroy D.C., Selzer M., Lokeshwar B., Pflugfelder S.C. (1999). Tear fluid gelatinase B activity correlates with IL-1α concentration and fluorescein clearance in ocular rosacea. Investig. Ophthalmol. Vis. Sci..

[41] Chotikavanich S., de Paiva C.S., Chen J.J., Bian F., Farley W.J., Pflugfelder S.C. (2009). Production and activity of matrix metalloproteinase-9 on the ocular surface increase in dysfunctional tear syndrome. Investig. Ophthalmol. Vis. Sci..

[42] Pflugfelder S.C., Farley W., Luo L., Chen L.Z., de Paiva C.S., Olmos L.C., Li D.-Q., Fini M.E. (2005). Matrix metalloproteinase-9 knockout confers resistance to corneal epithelial barrier disruption in experimental dry eye. Am. J. Pathol..

[43] Kimura K., Teranishi S., Fukuda K., Kawamoto K., Nishida T. (2008). Delayed disruption of barrier function in cultured human corneal epithelial cells induced by tumor necrosis factor-α in a manner dependent on NF-κB. Investig. Ophthalmol. Vis. Sci..

[44] Sternlicht M.D., Werb Z. (2001). How matrix metalloproteinases regulate cell behavior. Annu. Rev. Cell Dev. Biol..

[45] Tsubota K., Yokoi N., Shimazaki J., Watanabe H., Dogru M., Yamada M., Kinoshita S., Kim H.-M., Tchah H.-W., Hyon J.Y. (2017). New perspectives on dry eye definition and diagnosis: A consensus report by the Asia Dry Eye Society. Ocul. Surf..

[46] Pflugfelder S.C., Bian F., De Paiva C. (2017). Matrix metalloproteinase-9 in the pathophysiology and diagnosis of dry eye syndrome. Metalloproteinases Med..

[47] Gipson I.K., Hori Y., Argüeso P. (2004). Character of ocular surface mucins and their alteration in dry eye disease. Ocul. Surf..

[48] Kirschweng B., Tilinger D.M., Hégely B., Samu G., Tátraaljai D., Földes E., Pukánszky B. (2018). Melt stabilization of PE with natural antioxidants: Comparison of rutin and quercetin. Eur. Polym. J..

[49] de Queiroz K.B., Pereira T.d.S.F., Araújo M.S.S., Gomez R.S., Coimbra R.S. (2018). Resveratrol Acts Anti-Inflammatory and Neuroprotective in an Infant Rat Model of Pneumococcal Meningitis by Modulating the Hippocampal miRNome. Mol. Neurobiol..

[50] Zhu X., Huang F., Xiang X., Fan M., Chen T. (2018). Evaluation of the potential of chicoric acid as a natural food antioxidant. Exp. Ther. Med..

[51] Romero-Pérez A.I., Ibern-Gómez M., Lamuela-Raventós R.M., de la Torre-Boronat M.C. (1999). Piceid, the major resveratrol derivative in grape juices. J. Agric. Food Chem..

[52] Li X.-H., Gong X., Zhang L., Jiang R., Li H.-Z., Wu M.-J., Wan J.-Y. (2013). Protective effects of polydatin on septic lung injury in mice via upregulation of HO-1. Mediat. Inflamm..

[53] Indraccolo U., Barbieri F. (2010). Effect of palmitoylethanolamide–polydatin combination on chronic pelvic pain associated with endometriosis: Preliminary observations. Eur. J. Obstet. Gynecol. Reprod. Biol..

